# Photobiomodulation Mediates Neuroprotection against Blue Light Induced Retinal Photoreceptor Degeneration

**DOI:** 10.3390/ijms21072370

**Published:** 2020-03-30

**Authors:** Nora Heinig, Ulrike Schumann, Daniela Calzia, Isabella Panfoli, Marius Ader, Mirko H. H. Schmidt, Richard H. W. Funk, Cora Roehlecke

**Affiliations:** 1 Institute of Anatomy, Medical Faculty Carl Gustav Carus, Technische Universität (TU) Dresden, School of Medicine, Dresden 01307, Germany; ulrike.schumann@tu-dresden.de (U.S.); mhhs@mailbox.tu-dresden.de (M.H.H.S.); richard.funk@tu-dresden.de (R.H.W.F.); cora.roehlecke@tu-dresden.de (C.R.); 2 Department of Pharmacy-DIFAR, Biochemistry and Physiology Lab., University of Genoa, Genova 16132, Italy; dcalzia@gmail.com (D.C.); panfoli@difar.unige.it (I.P.); 3 Center for Regenerative Therapies Dresden (CRTD), TU Dresden, Dresden 01307, Germany; marius.ader@tu-dresden.de; 4 German Cancer Consortium (DKTK), Partner Site Dresden 01309, Germany; 5 German Cancer Research center (DKFZ), Heidelberg 69120, Germany

**Keywords:** low-level laser therapy, red light, near-infrared light, photoreceptor survival, respiratory chain complexes, oxidative stress, α-Crystallins, neuroprotection

## Abstract

Potent neuroprotective effects of photobiomodulation with 670 nm red light (RL) have been demonstrated in several models of retinal disease. RL improves mitochondrial metabolism, reduces retinal inflammation and oxidative cell stress, showing its ability to enhance visual function. However, the current knowledge is limited to the main hypothesis that the respiratory chain complex IV, cytochrome c oxidase, serves as the primary target of RL. Here, we demonstrate a comprehensive cellular, molecular, and functional characterization of neuroprotective effects of 670 nm RL and 810 nm near-infrared light (NIRL) on blue light damaged murine primary photoreceptors. We show that respiratory chain complexes I and II are additional PBM targets, besides complex IV, leading to enhanced mitochondrial energy metabolism. Accordingly, our study identified mitochondria related RL- and NIRL-triggered defense mechanisms promoting photoreceptor neuroprotection. The observed improvement of mitochondrial and extramitochondrial respiration in both inner and outer segments is linked with reduced oxidative stress including its cellular consequences and reduced mitochondria-induced apoptosis. Analysis of regulatory mechanisms using gene expression analysis identified upregulation *α-crystallins* that indicate enhanced production of proteins with protective functions that point to the rescued mitochondrial function. The results support the hypothesis that energy metabolism is a major target for retinal light therapy.

## 1. Introduction

Stimulation with far red light (RL) to near-infrared light (NIRL) in a range of 600–1000 nm with low intensities, called photobiomodulation (PBM), is described as a possible non-invasive strategy for additive therapy in retinal disorders. It has been used over many years for a range of clinical applications, including chiropractic, dental and dermatologic disorders and central nervous system injury [1]. A range of applications have shown beneficial effects in tissue injury, for example wound healing [2,3] improved neurological function including retinal degeneration, optic nerve injury, traumatic brain injury and spinal cord injury [4,5,6,7,8]. Various studies have focused on PBM effects such as cell proliferation and migration, oxidative stress, apoptosis and inflammatory processes in different disease models [9].

Although PBM shows positive effects, the underlying cellular and molecular mechanisms are only poorly described, especially concerning biological reactions. Most studies focused on cytochrome c oxidase (CCO) serving as primary photoacceptor and being the key target and effector for PBM, leading to enhanced energy metabolism [10,11,12]. CCO, complex IV in the mitochondrial respiratory chain, functions as a chromophore, which contains heme and copper centers to absorb light in the red to near-infrared spectral range.

In recent years, there has been an increasing interest in using PBM for therapy of retinal diseases. Protective functions were reported for different neuronal and nonneuronal retinal cell types, i.e., photoreceptors, the retinal pigment epithelium (RPE), retinal ganglion cells and Müller cells [4,13,14,15,16,17,18,19,20]. Besides studying individual cell types, several studies focus on effects in different diseases models such as age-related macular degeneration (AMD) and retinopathy models for possible transfer into therapy [18,21,22,23,24,25,26]. Most of these retina-related studies concentrate especially on the beneficial effects of 670 nm red light on retinal function, while other wavelengths are only rarely studied [4,27]. Results support the hypothesis of CCO as the primary target of the mitochondrial respiratory chain leading to enhanced retinal ATP production in AMD models [21,24,25,28] and increased mitochondrial membrane potential, shown in murine RPE [26].

In addition to mitochondrial components, retina-related studies demonstrate positive RL effects concerning retinal inflammation and cell stress in models of AMD. In particular, tumor necrosis factor-α, calcitonin, C3 and 4-hydroxy-2-nonenal (4HNE) were reduced in the outer retina and C3b, C3d and amyloid-beta expression decreased in Bruchs membrane [18,21,26]. Moreover, reduced neovascularization and reduced photoreceptor cell death in oxygen-induced retinopathy models were reported [18,21,22,23]. Additionally, the protection of the photoreceptor ultrastructure combined with an improved retinal function verified by electroretinogram were demonstrated in an animal model with methanol induced mitochondrial dysfunction [6]. Only very few publications studied the ability of wavelengths other than 670 nm to be supportive for retinal injuries. Giacci et al. analyzed effects of 670 nm and 830 nm on a retinal degeneration model, showing reduced cell death and 8-hydroxyguanosine immunoreactivity after 670 nm pretreatment, but no changes in 830 nm treated animals [4]. However, Ivandic et al. observed positive effects of 780 nm light treatment; they reported improved visual acuity, reduced edema and blebbing in wet AMD [27]. The addressed RL and NIRL studies indicate that PBM can offer a non-invasive approach that is easy to deliver, to prevent or slow down the progress of retinal pathology.

Focusing on collective PBM effects is a demanding question since a large number of studies use different irradiation sources, different wavelengths, durations and frequencies. Therefore, we decided to analyze two different wavelengths (670, 810 nm) with different irradiation sources (LED and diode laser) to reach a higher consensus and comparability.

The purpose of the present study was to examine whether PBM has beneficial effects in light-induced photoreceptor damage to achieve a better understanding of the underlying cellular and molecular mechanisms of RL and NIRL action. To verify beneficial effects on photoreceptors, the oxidative cell stress was analyzed by measuring reactive oxygen species (ROS), lipid peroxidation and mitochondria-related apoptosis. Due to the recommended action of PBM on mitochondrial function on oxidative cell stress and cell viability, the participation of RL and NIRL action on respiratory complexes was analyzed. Currently, there is limited knowledge concerning the influence of PBM on respiratory chain complexes, mainly focusing on complex IV, leading to improved cell survival. Therefore, the additional analysis of other complexes might explain the connections of the RL and NIRL action more conclusively. Moreover, the RL and NIRL triggered regulatory effects based on mRNA alterations were examined, which might explain these observations.

To examine the positive effects of PBM, an established ex vivo retina tissue cultivation system was used [29,30]. With regard to our study, an ex vivo application, especially whole eyeballs cultivation, is favorable as it combines the efficiency and control common to in vitro techniques with close imitation of the in vivo environment. Ex vivo models are common experimental designs to examine retinal tissue regeneration with a highly controlled setting, e.g., by the delivery of equal amounts of light to the retina by a constant distance [29,31,32]. We chose blue light (BL) irradiation as a damaging model of irradiated photoreceptors and analyzed cellular and molecular action of RL or NIRL after post-treatment [29,30]. The exposure to intense artificial light (e.g., white LED) with enriched emission spectrum in blue radiations and blue light is a risk factor causing photochemical damage. BL leads to oxidative stress in cells, at first observed in the photoreceptor cells. Due to their high content of mitochondria, they are especially susceptible to oxidative stress. Pathological effects of light-damaged photoreceptors are the structural degeneration of outer segments (OS), oxidative stress, lipid oxidation and cell death [29,30,33].

Altogether, we report for the first time a complex pattern of beneficial neuroprotective effects by red and near-infrared light on BL-damaged photoreceptors that are valuable for transferring experimental RL/NIRL therapy into clinical applications.

## 2. Results

An established ex vivo BL retina damaging model that mimics photochemical damaging was used to evaluate the cellular and molecular mechanisms of RL and NIRL [29,30].

**Figure 1:**
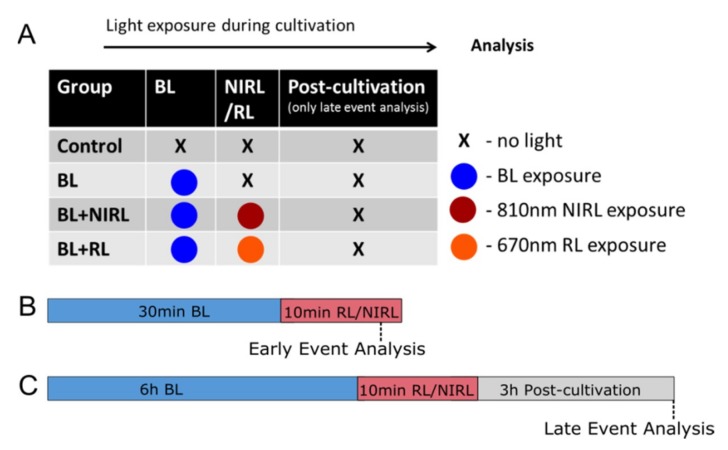
Schematic set up of mouse eyes cultivation with light irradiation. (**A**) Eyes were grouped regarding their exposure to light as following; Control: Non-light-irradiated eyes; BL: Only blue light irradiated eyes; BL+NIRL 810 nm: blue light irradiation + near-infrared light postexposure 810 nm diode laser; BL+RL 670 nm: blue light irradiation + red light postexposure 670 nm LED. (**B**) Early event analysis after 40 min of cultivation. Eyes were treated for 0.5 h with BL, following 10 min NIRL or RL and direct analysis after light treatment. Only reactive oxygen species (ROS) analysis using CM-H_2_DCFDA vital dye was performed after 40 min of cultivation. (**C**) Late event analysis was performed after 9 h of cultivation. Eyes were treated for 6 h with BL, following 10 min NIRL or RL and post-cultivated for another 3 h.

In order to be able to use ex vivo cultivation as a model of damage, it had to be ensured beforehand that cultivation itself does not cause significant morphological retinal damage. The issue was addressed by HE-staining of cultivated retina, resulting in an optimal total cultivation time of 9 h (Figure S1). This blue light model is especially characterized by oxidatively stressed photoreceptors [29,30]. Figure S2 in the supplement shows apoptotic cells in untreated and blue light damaged retinas, confirming that photoreceptors are the first cell type where oxidative stress due to blue light irradiation occurs, whereas retinal cells in the ganglion cell layer (GCL) and inner nuclear layer (INL) are not affected. To examine the impact of RL and NIRL stimulation on BL-irradiated photoreceptors, eyes were damaged with blue light followed by RL/NIRL treatment. Eyes were grouped regarding their exposure to light (Figure 1).

### 2.1. Decreased Mitochondria-Induced Apoptosis upon RL/NIRL Exposure

**Figure 2:**
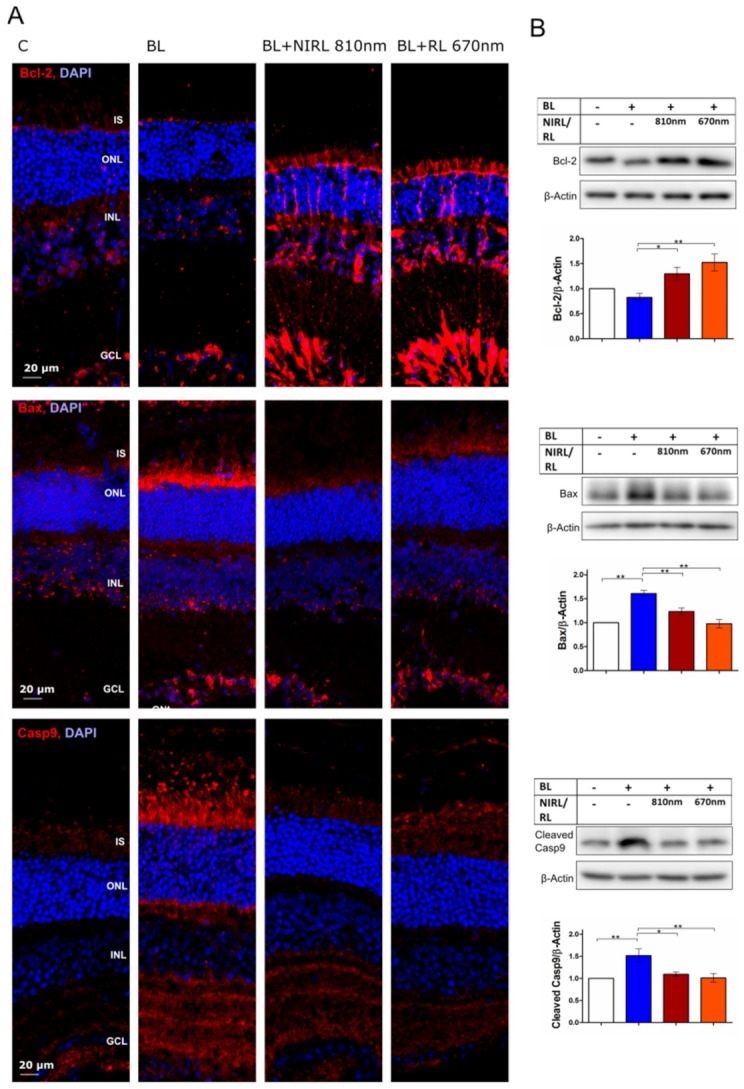
NIRL and RL inhibit mitochondria induced apoptosis pathway through Bcl-2, Bax and Caspase-9 regulation. (**A**,**B**) Late event analysis after 9 h of cultivation. Eyes were treated for 6 h with BL, following 10 min NIRL or RL and post-cultivated for another 3 h. (**A**) Representative images of Bcl-2, Bax and Caspase-9 (Casp9) immunohistochemical staining in retinal sections. Retinal layers: outer segments [OS], inner segments [IS], outer nuclear layer [ONL]. *N* = 3 experiments. (**B**) Western blot analysis of retinal lysates using antibodies against Bcl-2, Bax and Caspase-9 and β-Actin as loading control. Protein level was quantified using ImageJ. *N* = 6 experiments ± standard error of the mean (SEM). * *p* < 0.05, ** *p* < 0.01 compared with blue light.

To verify the effects of RL and NIRL on elements of the intrinsic mitochondria-induced apoptotic pathway in photoreceptors, Casp9, Bcl-2 and Bax protein expressions were analyzed. Upon BL irradiation protein expression of pro-apoptotic Bax and Caspase-9 was significantly increased in photoreceptor inner segments (IS) compared to non-irradiated controls, while Bcl-2 remained unchanged. Compared to only BL irradiated photoreceptors, the Bax and Caspase-9 protein levels decreased significantly after RL or NIRL treatment in mitochondria-rich IS. In contrast, anti-apoptotic Bcl-2 protein expression increased in IS upon RL or NIRL exposure (Figure 2A). Aside from the localization in the IS, Bcl-2 expression was observed in RL- or NIRL-treated retinas in retinal ganglion cells and in Müller cells (Figure 2A and Figure S3). In conformity with the immunohistochemical staining, analyzed western blots show a significantly lowered protein expression of Bax and Caspase-9 and increased protein expression of Bcl-2 after exposure to 810 nm NIRL and 670 nm RL (Figure 2B). Data indicate less mitochondria-induced apoptosis upon RL or NIRL treatment compared to BL damaged photoreceptors. Additionally, a terminal deoxynucleotidyl transferase (TdT) dUTP Nick-End Labeling (TUNEL) assay and Caspase-3 immunohistochemical staining support the observation of increased photoreceptor survival after RL or NIRL treatment (Figure S4).

### 2.2. Increased Respiration upon RL/NIRL Exposure

**Figure 3:**
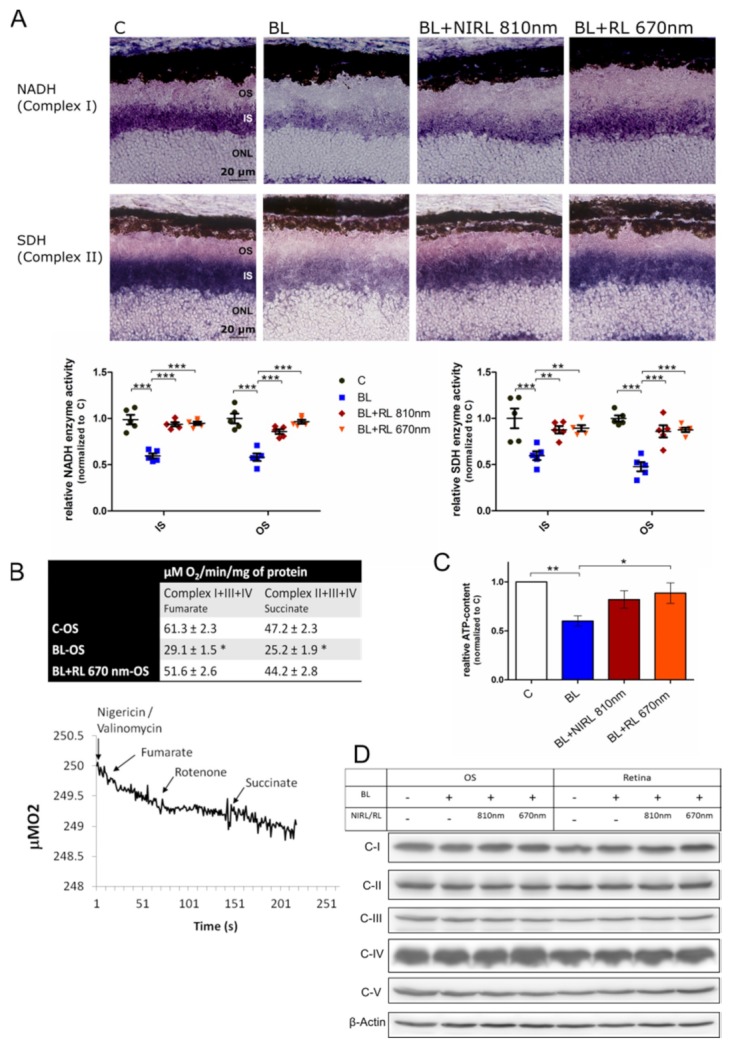
NIRL and RL increase oxidative phosphorylation in BL irradiated cells. (**A**–**D**) Late event analysis after 9 h of cultivation. Eyes were treated for 6 h with BL, following 10 min NIRL or RL and post-cultivated for another 3 h. (**A**) Representative images of NADH (Complex I) and SDH (Complex II) enzyme activity, labeled on unfixed retinal cryo-sections. Staining represents reduction of NBT to NBT-formazan catalyzed by oxidation of NADH or SDH. Retinal layers: outer segments [OS], inner segments [IS], outer nuclear layer [ONL]. The graph displays quantitative analysis of NADH and SDH relative enzyme activity: By measuring grey values of IS and OS the relative enzyme activity was calculated and normalized to Control (C). *N* = 5 experiments ± SEM. (**B**) Table shows oxygen consumption rates in purified OS (0.04 mg protein) for each of the two pathways: OXPHOS I+III+IV and OXPHOS II+III+IV. To stimulate the first pathway 20 mM fumarate was employed and 20 mM succinate for the second pathway. Additionally, a representative amperographic respiratory trace of Control OS assayed in the presence of substrates specific to the pathway Complex I+III+IV (Fumarate) or the pathway Complex II+III+IV (Succinate) is shown. *N* = 6 experiments ± SEM. * *p* < 0.05 BL compared with Control and BL+RL 670 nm-OS. (**C**) ATP-content analysis of retina lysates using ATP bioluminescence assay. The graph displays mean values normalized to Control of *n* = 7 experiments ± SEM. (**D**) Western blot analysis of retina lysates and isolated OS using OXPHOS antibody mix (Complex I: NDUFB8, Complex II: SDHB, Complex III: UQCRC2, Complex IV: MTCOI, Complex V: ATP5A) shows no significant differences in protein expression. β-Actin served as loading control (*n* = 4 experiments). * *p* < 0.05, ** *p* < 0.01, *** *p* < 0.001 compared with BL.

To study the activity of oxidative phosphorylation (OXPHOS), we examined photoreceptors after RL and NIRL exposure regarding respiration. A histochemical assay on unfixed sections was performed to determine the activity and the localization of the OXPHOS complexes. Complex I (NADH-CoQ oxidoreductase) and II (Succinate dehydrogenase, SDH) were chosen, as these represent the starting points of the two OXPHOS pathways (complex I+III+IV or complex II+III+IV). While OXPHOS is closely associated with mitochondria, recently, functional extramitochondrial complexes have also been described within outer segments (OS) that are devoid of mitochondria. Thus, OXPHOS activity was expected in the mitochondria-rich IS as well as in the OS of photoreceptors [34]. Upon BL irradiation the activity of complex I decreased drastically by 0.40 in the mitochondria rich IS and by 0.42 in OS, respectively, related to normalized control. Complex II activity was reduced similarly by 0.40 in IS and by 0.52 in OS. In contrast to only BL exposed samples, the BL+RL and BL+NIRL treated photoreceptors restored their enzyme activity close to control levels (Figure 3A). Increased complex activities upon RL or NIRL exposure were observed in the mitochondria-rich IS but also in the OS. Upon 670 nm RL exposure Complex I activity increased in IS to 0.95 and in OS to 0.97. After 810 nm NIRL exposure, Complex I activity increased in IS to 0.94 and in OS to 0.88. Complex II activity was restored as well, being increased to 0.89 in IS and to 0.88 in OS upon 670 nm RL exposure and to 0.88 and to 0.86 after 810 nm NIRL exposure in IS and OS, respectively, related to controls. In addition to the histochemical investigation, OXPHOS levels were further analyzed by measuring oxygen consumption on isolated OS. The OXPHOS pathway I+III+IV was addressed by stimulation with fumarate and the OXPHOS pathway II+III+IV by adding succinate. RL or NIRL stimulation recovered the BL lowered oxygen consumption, indicating an increased flux through the electron transport chain (Figure 3B). Oxygen consumption increased by 84% for complex I and by 94% for complex II, after irradiation with 670 nm RL compared to non-irradiated control (Figure 3B).

A possible increase of ATP synthesis as a consequence of improved mitochondrial respiration was assessed by analyzing intracellular ATP content of retinal lysates. As anticipated, the 670 nm RL treatment upregulated the ATP-content significantly, compared to BL conditions. The same trend was observed for the 810 nm NIRL treatment (Figure 3C). Interestingly, in contrast to the modulation of their activity, neither BL nor RL or NIRL stimulation altered the protein expression level of the OXPHOS complexes (Figure 3D).

### 2.3. Decreased ROS Levels after RL/NIRL Exposure

**Figure 4:**
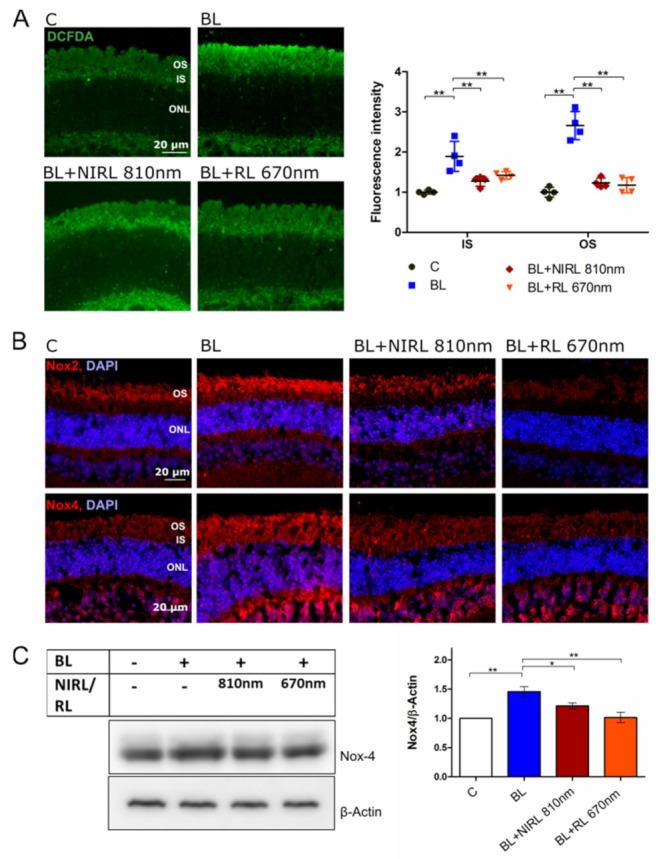
NIRL and RL induce reduced ROS production in BL irradiated cells. (**A**) Representative images of CM-H_2_DCFDA labeled unfixed retinal vibratome sections. Staining demonstrates ROS production in form of hydrogen peroxide (H_2_O_2_), peroxynitrite anions (ONOO^-^), hydroxyl radicals (HO^.^) or peroxide radicals (ROO^.^). Retinal layers: outer segments [OS], inner segments [IS], outer nuclear layer [ONL]. The graph displays fluorescence intensity of IS and OS normalized to Control (IS), demonstrating ROS production of *n* = 4 experiments ± SEM. Retinas were cultivated for 40 min (Control: 40 untreated, BL: 30 min BL + 10 min post-cultivation, BL+NIRL 810 nm: 30 min BL + 10 min 810 nm RL, BL+RL 670 nm: 30 min BL + 10 min 810 nm NIRL) for early event analysis. (**B**,**C**) Late event analysis after 9 h of cultivation. Eyes were treated for 6 h with BL, following 10 min NIRL or RL and post-cultivated for another 3 h. (**B**) Representative images of Nox2 and Nox4 immunohistochemical staining in retinal sections. *N* = 3 experiments. (**C**) Western blot analysis of isolated OS using antibodies against Nox4 and β-Actin as loading control. Protein level was quantified using ImageJ. *N* = 6 experiments ± SEM. * *p* < 0.05, ** *p* < 0.01 compared with BL.

As oxidative stress and dysfunction of oxidative phosphorylation can cause increased reactive oxygen species (ROS) production, ROS levels were assessed by analyzing vital dye 2′,7′–dichlorofluorescin diacetate (DCFDA) and indirectly by NADPH oxidases Nox2 and Nox4 (Figure 4). Firstly, the level of ROS fluorescence intensity in BL irradiated eyes increased in IS from 1 to 1.89 and in OS from 1 to 2.66 in comparison to normalized control (Figure 4A). Secondly, upon RL/NIRL exposure, an immense reduction of ROS levels were detectable in both IS and OS. Following 810 nm NIRL or 670 nm RL stimulation, the ROS level decreased by 0.63 (810 nm NIRL) or 0.50 (670 nm RL) in IS and by 1.43 (810 nm NIRL) or 1.49 (670 nm RL) in OS, achieving levels close to control.

Protein expression of Nox2 and Nox4 increased upon BL exposure, but decreased in photoreceptor IS and OS after RL or NIRL irradiation (Figure 4B). Quantification via western blot showed a significant reduction of Nox4 expression in isolated OS compared to only BL irradiated samples, although retinal Nox4 gene expression did not significantly change, which suggests posttranslational modifications (Figure 4C and Figure S5).

We suggest that the significantly decreased intracellular production of free radicals is particularly important considering the high polyunsaturated fatty acids (PUFAs) content in photoreceptors. To investigate the effect on lipid peroxidation, we utilized the generation of highly electrophilic aldehydes 4HNE and N-epsilon-(hexanoyl)lysine (HEL). Indeed, we observed a positive effect of RL and NIRL exposure for lipid peroxidation. HEL and 4HNE expression decreased in both IS and OS of BL+NIRL 810 nm and BL+RL 670 nm samples (Figure S6).

### 2.4. RL/NIRL Regulated the Expression of Genes Associated with Protective Functions

**Figure 5:**
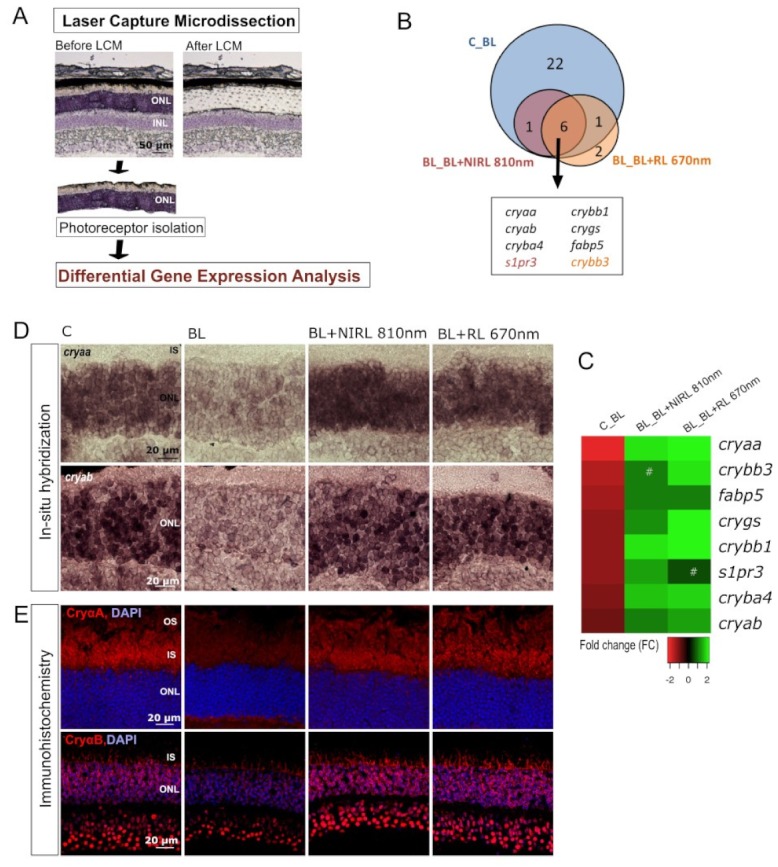
NIRL and RL regulated gene expression analysis of isolated photoreceptors. (**A–E**) Late event analysis after 9 h of cultivation. Eyes were treated for 6 h with BL, following 10 min NIRL or RL and post-cultivated for another 3 h. (**A**) Schematic set up of microdissected photoreceptors via Laser Capture Microdissection (LCM) for gene expression analysis. (**B**) Venn diagram illustrating overlap of significant differentially expressed genes (DEGs) from C_BL, BL_BL+NIRL 810 nm and BL_BL+RL 670 nm with a cutoff of log_2_ FC > 0.5 and an adjusted p-value < 0.1 (**C**) Heatmap of overlapping up- and downregulated genes significant for C_BL and BL_BL+NIRL 810 nm or BL_BL+RL 670 nm ranked by fold change. *N* = 2 experiments. #—not significant. (**D**) Representative images for situ hybridization from *cryaa and cryab* mRNA in retinal sections. *N* = 3 experiments E: Representative images of CryαA and CryαB immunohistochemical staining in retinal sections. D, E: Retinal layers: outer segments [OS], inner segments [IS], outer nuclear layer [ONL]. *N* = 3 experiments.

To gain more insight into the regulatory factors that mediate modifications of photoreceptors due to RL and NIRL, we performed mRNA gene expression analysis of isolated photoreceptors. Since only microdissected photoreceptors from whole retinas were utilized, the observed changes in gene expression could not be compensated or altered by other cell types of the retina (Figure 5A). Major up- and downregulated genes were observed between three different clusters: C vs. BL (C_BL), BL vs. BL+NIRL 810 nm (BL_BL+NIRL 810 nm) and BL vs. BL+RL 670 nm (BL_BL+RL 670 nm). In total, the RNA sequencing identified 35,169 annotated genes, with significantly altered expression of 22 genes for C_BL, 7 genes for BL_BL+NIRL 810 nm and nine genes for BL_BL+RL 670 nm (Figure 5B and Table S1). Differential gene expression profiles of each separate comparison with a cutoff of log_2_ FC > 0.5 are illustrated in volcano plots (Figure S7). To outline the effect of RL, eight overlapping differentially expressed genes (DEGs) from C_BL, BL_BL+NIRL 810 nm and BL_BL+RL 670 nm are shown in Figure 5B,C, including a heatmap demonstrating their fold change. The overlapping DEGs show strong evidence that especially different genes of *crystallin* classes are altered by BL and RL/NIRL treatment. Upon RL or NIRL exposure *crystallin* genes were upregulated 1.1–2.2-fold compared to BL irradiated photoreceptors. Of particular interest were *αA-crystallin* and *αB-crystallin* (*cryaa* and *cryab)*, for which distinct retinal functions have been identified, for example anti-apoptotic and anti-oxidative functions [35,36].

## 3. Discussion

While RL/NIRL treatment is already in clinical use, including the application for retinal disorders, the molecular mechanisms underlying its therapeutic effect are not yet fully known. Consequently, in this study, we addressed the neuroprotective activities of 670 nm RL and 810 nm NIRL against BL toxicity in photoreceptors focusing on the molecular implications of RL/NIRL. Several studies showed that stress markers and inflammatory markers in diseased retinas can be reduced by 670 nm red light exposure in the outer retina. RL is selectively absorbed by CCO of mitochondria; however, the exact mechanism or impact on transcription factors and gene expression is largely unknown [21,24,25,28]. While authors postulate CCO to be the main target of RL action leading to improved mitochondrial function and thus reduced cell death, there is still limited information about the impact of RL/NIRL on other respiratory complexes or mitochondria-induced apoptosis in retinal diseases. In detail, we examined the ability of RL and NIRL to reduce oxidative damage and cell death, as well as the impact on the respiratory chain complexes and regulatory mechanisms based on differentially expressed genes that might explain the observed effects on protein level.

Our data concerning the role of intrinsic mitochondria-driven apoptotic pathways as well as those of Gu et al. show that light-induced retinal damage particularly stresses mitochondria, leading to cytochrome c release into the cytosol triggering apoptosis [37]. Here, we demonstrate for the first time a decreased Caspase-9 expression in mitochondria-rich IS, suggesting reduced mitochondria related apoptosis of RL/NIRL stimulated photoreceptors. Only for visual cortical neurons, an effect of 670 nm RL on Caspase-9 was shown before [38]. The speculated increased loss of cytochrome c after BL damage from both IS mitochondria and OS disks is probably responsible for the reported increased rod apoptosis, in turn triggering the retinal damage [39]. Ghafourifar et al. described a lack in transferring electrons from complex III to IV that arises through membrane opening and the release of cytochrome c into the cytosol leading to reduced respiration rates in cells [40].

Few studies showed the effect of PBM on mitochondria induced apoptosis focusing on Bcl-2, Bcl-x_l_ and Bax. These studies were performed using PC12 cells [41], skeletal muscle cells [42] and visual cortical neurons [38]. They confirm our results of increased cell survival protein Bcl-2 and decreased cell death protein Bax. Altered Bax and Bcl-2 expression strongly indicate that RL/NIRL lowers membrane channel formation, causing a smaller amount of cytochrome c release, ultimately preventing BL irradiated cells undergoing mitochondria related apoptosis. The inhibitory effect of RL/NIRL on cell apoptosis goes hand in hand with increased Bcl-2 and decreased Bax expression.

In particular, RL exposure reverted Bax and Caspase-9 after a 50% increase caused by BL treatment, while increasing Bcl-2 by 50%, as compared to controls. This may appear inconsistent with the fact that BL reduces mitochondrial ATP, necessary to protein synthesis. One hypothesis is that in case of damage ATP is diverted towards its use for the synthesis of proteins that help the cell to avoid the injury. Due to blue light stress conditions, ATP molecules might be captured for the synthesis of stress related proteins, i.e., Bax or survival proteins. This BL-triggered cell reaction might lead to a reduced total amount of ATP. Further ATP production may not be sufficient due to inhibition of respiratory chain complexes. Another intriguing possibility is that the ATP content of the retinal lysates is mainly affected by the absolute content of ATP in the rod OS, which displays a considerable ATP synthetic ability (about 0.6 micromoles/min/mg of protein) [43]. Consequently, the total ATP content of the retinas would not be representative of the actual fluctuations in ATP content of retinal components other than the OS. Moreover, the measurement of total ATP cannot represent its nano-local turnover fluctuations.

The rod OS cannot carry out protein synthesis, being devoid of ribosomes and DNA: consistently neither BL nor RL stimulation altered the protein expression level of the OXPHOS complexes. Along with this view, it is conceivable that RL increases Bcl-2 by 50% compared with controls and that BL increases Bax by 50%, as data show, regardless of the measures of total ATP. On the other hand, an experimental time of 6 h and 9 h is sufficient to involve protein synthesis in those parts of the retina that can actually conduct it.

Most studies regarding PBM postulate that the mechanism of 670 nm RL irradiation rests on photon absorption by mitochondrial complex IV, leading to enhanced ATP production and mitochondrial function in general [21,24,25,28]. Our results confirm that 670 nm RL, but also the less analyzed 810 nm NIRL photo-stimulates mitochondrial activity. We observed that 670 nm RL and 810 nm NIRL treatment increased the activity of complexes I and II, i.e., the two OXPHOS pathways complex I+III+IV and complex II+III+IV in photoreceptor IS and OS and retinal ATP production after a drastic inhibition through BL irradiation.

In comparison to former studies on RL/NIRL, our results demonstrate the participation of additional complexes I and II besides the proposed photoacceptor CCO [12,21,24,25,28,44]. Our results indicate that CCO may not be the main target of PBM action, leading to reduced oxidative stress. The observed upregulation of complex I and II activity can implicate improved CCO activity by direct or indirect interaction between the complexes. Either CCO is directly addressed by RL/NIRL or it is a secondary effect by prior activation of complexes I and II. The second hypothesis is supported by our results, showing no increase of CCO protein content upon PBM treatment. An indirect interaction between complex I and CCO is conceivable through the formation of Bax/complex I after blue light damage that is dissociated by high levels of pro-survival Bcl-2 family proteins after RL/NIRL treatment. The Bax/complex I interaction would down regulate the respiratory chain activity via p53 [45].

In line with our suggestions, the data from some studies indicate the hypothesis that CCO is not, as is broadly assumed, the main target of PBM. For example, Amaroli et al. described that the activity of complex III is increased by 808 nm NIRL. Though complex IV is increased, I and II are not affected [46]. In a different study, Yu et al. demonstrated enhanced complex I, III, IV activity in rat liver mitochondria [47]. Still, these studies of isolated bovine or rat liver mitochondria represent a different milieu than our study of complete retinal explants with physiological cellular environment, which may cause an altered response of the complexes. Definitely, the determined activation of complexes I and II results in an increased flux through electron transport chain pathways by causing improved activity of the complexes III and IV and an increased ATP-content, either in direct or indirect interaction with the complexes.

One recent study determined that CCO is not necessary for PBM effects by describing an enhanced proliferation of cells lacking CCO [48]. In addition, Mason et al. suggested appropriate caution about data interpretation of the mechanism of action of PBM on CCO due to large changes in the population of the oxygen intermediates in CCO [49]. They proposed that either cupric Cu_A_ center or oxidized heme a3/Cu_B_ binuclear center act as contributors of PBM depending on the RL/NIRL spectrum. However, they did not regard CCO as a target of RL protective action. In summary, studies for a final assessment whether CCO is affected as the main target during PBM are missing.

Not only the mitochondria-rich IS, but also the extramitochondrial complexes in OS are stimulated by RL/NIRL. The detected complex I and II activity in the mitochondria-deficient OS is consistent with functional extramitochondrial complexes in OS reported by Calzia et al. [50].

In conformity with our results, an increased intracellular ROS production in BL damaged cells was already proven [30,51]. When mitochondria are intact and in coupled status, increased oxygen consumption is associated with ATP synthesis. An increment in mitochondrial complex activities and oxygen consumption in damaged conditions can result in oxidative stress and ROS production [52]. Our results showing the restoration of BL-impaired respiratory capacity and of ATP content after RL or NIRL stimulation, support enhanced mitochondrial functioning, thus lowering oxidative cell stress.

The impact of RL/NIRL on ROS generation is inconsistent, as some found a reduction in ROS upon PBM, while others reported that ROS was, in fact, upregulated [16,17,53,54]. It is mentioned that RL/NIRL triggers moderately increased ROS and mediates its protective effect via the activation of the redox-sensitive transcription factor NF-ĸB [54]. Still, from the current literature, the mechanism for ROS production upon RL/NIRL stimulation is not fully understood, in addition to the cellular mechanisms responsible for balancing the ROS levels required for maintaining optimal mitochondrial function. The effect might depend on the type of cells, the damage status of the cell and the damaging model. It could, therefore, be concluded that too much ROS production causes damage but a moderate amount of ROS is not harmful, sometimes even protective. Consistent with what has been previously reported, the levels ROS staining in BL irradiated eyes were higher in OS than IS [52]. Our results show reduced ROS production in photoreceptor IS and OS upon RL/NIRL irradiation supporting the theory of less cell stress due to reduced intracellular ROS. However, the slight increase of ROS production of RL/NIRL treated compared to non-irradiated photoreceptors might be beneficial in activating NF-ĸB.

As the mitochondrial respiratory chain is the major ROS generation site, large amounts of ROS can be produced based on incomplete oxygen reduction by OXPHOS complexes of mitochondria [55]. We suggest that upon BL damage oxidative phosphorylation is uncoupled, leading to a sensibly reduced ATP synthesis and enhanced ROS production [52]. The detected extramitochondrial complexes might be an additional source for the production of free radicals besides the detected NADPH oxidases in OS [30]. The described membrane opening during mitochondrial related apoptosis pathway can also uncouple oxidative phosphorylation causing inhibition of ATP production and incomplete oxygen reduction shunted into ROS production [56].

Consistent with the modulatory effect from RL/NIRL stimulation on ROS production, the amount of detected lipid peroxidation in photoreceptors was reduced. In addition to the already reported 670 nm RL triggered reduction of 4HNE in photoreceptor OS and in the optic nerve, we could show a reduced HEL expression [4,18]. As mentioned, since most PBM studies are limited to 670 nm, we provide data about 810 nm NIRL exposure on 4HNE and HEL. We speculate that RL inhibits lipid peroxidation because of increased Bcl-2 expression. Bcl-2 overexpression leads to the complete suppression of lipid peroxidation [57]. RL/NIRL treatment modulation of ROS production and Nox4 expression consequent to BL irradiation was especially evident in isolated OS. Finally, the RL/NIRL mediated neuroprotective impact on ROS production and lipid peroxidation on photoreceptors leading to less oxidative cell stress.

One major point in our study was the analysis of regulatory factors that might mediate changes in oxidative stress levels and mitochondrial function upon BL and especially NIRL/RL treatment.

Our study revealed 22 genes with significant expression changes for C vs. BL, seven genes for BL vs. BL+NIRL 810 nm and nine genes for BL vs. BL+RL 670 nm. A recently published study identified gene expression changes in photoreceptors of *Drosophila melanogaster* [58]. The expression of 44 genes altered one day after BL expression and 568 genes 6 days after BL expression. These genes are part of stress response pathways, calcium influx and ion transport. This study, as well as our study, identified different DEGs, but both include genes with neuroprotective functions. Differences may have various causes, for example, the chosen species and BL exposure settings (duration, wavelength). Similar to the drosophila study, it might be possible that the small number of altered genes could increase after longer cultivation time or BL, RL and NIRL exposure time. Considering the mentioned study, we suggest that the identified DEGs are limited to the precise experimental conditions. Another possible explanation for a small number of DEGs considers that BL, RL and NIRL affect predominantly the protein level of murine photoreceptors.

The most common assumptions of RL/NIRL mediated alterations in genes include transcription factors such as NF-κB, RANKL, RUNX2 and HIF1α [59]. We were able to outline the changes of RL/NIRL on differential gene expression and identified genes that have potential neuroprotective functions. Our study showed that these significantly altered genes include *α-crystallins,* pointing to the rescued mitochondrial function. We demonstrate inhibited expression of *α-crystallins genes cryaa* and *cryab* after BL and upregulation upon RL stimulation. α-crystallins have already been described to be implicated in several cellular processes, including survival and cell death pathways, oxidative stress and neuroprotection. Our study provides evidence that PBM regulates the NF-κB pathway, which improves cell survival, via crystallins based on the recent discovery of NF-κB as a target of αB-crystallin [60]. Moreover, reported functions of α-crystallin in the retina and RPE indicate a role in mitochondria in an anti-apoptotic manner. The αB-crystallin is postulated to interact directly with the pro-apoptotic members Bax and Bcl-X_s_ suppressing mitochondrial apoptosis [36]. A deficiency of αB-crystallin increased the level of ROS and consequently cells exposed to oxidative stress exhibited significant changes in mitochondrial permeability transition and activation of Caspase-3 [61,62]. These studies support our findings of reduced oxidative stress and enhanced mitochondrial function triggered by RL. As α-crystallins are linked to cellular GSH levels, we assume participation of activating antioxidants through RL, in particular GSH [63]. Nevertheless, some studies report increased expression of α-crystallins in RPE and retina following light exposure as well [64,65]. However, distinct functions of α-crystallins are still under debate, as they can be pro- or anti-inflammatory in different retinal disease models [66,67,68,69,70]. Further investigation will be needed to elucidate the role of α-crystallins concerning PBM.

Our results provide an improved understanding of the multiple RL and NIRL-triggered neuroprotective defense mechanisms that allow photoreceptor rescue. Data show that the target of RL and NIRL is indeed the oxidative metabolism of the photoreceptor, similar to the BL-induced damage. RL and NIRL demonstrates neuroprotective activities to rescue the deadly consequences of OXPHOS impairment in terms of hypo-metabolism (i.e., low ATP content) and oxidative stress, in turn triggering apoptosis, caused either by Bax translocation on the mitochondrial membrane or cytochrome c exit from the IS mitochondria or the OS disks. This reveals a mechanism targeting the respiratory chain, both in their mitochondrial and extra-mitochondrial location. Our findings suggest the use of PBM as a noninvasive additive therapeutic treatment for numerous retinal diseases linked to mitochondrial dysfunction. The next step for transferring the results into a more therapeutic direction would be to initiate a follow-up study using an in vivo model.

## 4. Materials and Methods

### 4.1. Animals

All animal experiments were approved by the ethics committee of the TU Dresden and the license for removal of organs was provided by the Landesdirektion Dresden (Az.: 24D-9168.24-1/2007-27). All experiments were performed in accordance with relevant guidelines and regulations

### 4.2. Organ Culture and Irradiation with Light

On postnatal day 24±4, C57BL/6 mice of either sex were sacrificed by cervical dislocation. The eyes were cultivated according to the established organotypic model of photoreceptors [29,30].

To enable fluid exchange, the eyeballs were punctured with a needle to create a small hole and were transferred in a six-well plate filled with 1 mL DMEM/F12 medium per well containing 10% fetal calf serum, 2% B-27 supplement (Invitrogen; Carlsbad, CA, USA, 1% penicillin-streptomycin and 2 mM glutamine. To fixate the eye position, the eyes were positioned in a cell culture insert (Corning^®^ Transwell^®^ polyester membrane cell culture inserts 24 mm Transwell, 0.4 μm pore) in six-well plates, so that their corneas faced the light sources. The eyes were incubated at 37 °C, with a CO_2_ level of about 5% in a cell culture incubator (Figure S8).

After blue light (BL) irradiation, eyes were post-irradiated with red light (670 nm) or near-infrared light (810 nm) for 10 min. Next, the eyes were directly analyzed or further cultivated. The eyes were grouped based on their exposure to light and their total cultivation time (Figure 1A). For early event analysis, the eyes were irradiated for 30 min with BL, followed by 10 min RL or NIRL irradiation and direct analysis (Figure 1B). For late event analysis, the eyes were irradiated for 6 h with BL, followed by 10-min RL or NIRL irradiation and 3-h postcultivation (Figure 1C).

Blue light irradiation with a wavelength of 405 nm and an output power of 1 mW/cm^2^ was applied for 30 min delivering 1.8 J/cm^2^ of energy or for 6 h delivering 21.6 J/cm^2^, respectively. It was produced by an LED-based system (# LZ1-00UA05 BIN U8; LedEngin) that was constructed and established in our lab. RL illumination with a wavelength of 670 nm and an output power of 60 mW/cm^2^ was applied for 9 min, delivering 32.4 J/cm^2^ of energy. It was used repeatedly in six 90 sec treatments. NIRL illumination with a wavelength of 810 nm and an output power of 60 mW/cm^2^ was applied for 9 min delivering 32.4 J/cm^2^ of energy. RL was produced by an LED device (Warp10, Quantum Devices; Barneveld, WI, USA) and NIRL by a diode laser with Slit Lamp Adapter and Continuous-Wave-Mode (OcuLight^®^SLx, Iridex; Mountain View, CA, USA)). The used organ culture provides cells in a vital and reactive status and exhibits the advantage of uniform and optimally controlled conditions for light application.

### 4.3. Intracellular ROS Production

For evaluating ROS, we used CM-H_2_DCFDA (Molecular Probes^®^-Invitrogen, Carlsbad, CA, USA), an indicator of general ROS production in the form of hydrogen peroxide (H_2_O_2_), peroxynitrite anions (ONOO^-^), hydroxyl radicals (HO^.^), peroxide radicals (ROO^.^), superoxide (O_2_^·−^) or singlet oxygen (^1^O_2_). After cultivation, retinas were dissected and stained with 25 µM CM-H_2_DCFDA for 10 min at 37 °C. Samples were rinsed in PBS and fixed in 6% PFA for 0.5 h. After embedding in 4% agarose, the explants were cut in 40 µm vertical vibratome sections. Retinas were immediately analyzed using a Zeiss LSM 510 confocal laser scanning microscope. Same acquisition settings were used in all experiments and groups. The mean fluorescence intensities of OS and IS were determined in eight regions of interest (ROI), normalized to Control.

### 4.4. Measurement of ATP Content

The assessment of intracellular ATP content was performed utilizing ATP Bioluminescence Assay Kit HS II (Sigma-Aldrich; St. Louis, MI, USA) according to the manufacturer’s instructions and measured by a Tecan infinite M200 plate reader.

### 4.5. Histochemical Reactions for ETC I and II Activity

ETC I and II enzyme activity was analyzed using unfixed 14 µm tick cryosections. To analyze NADH Coenzyme Q oxidoreductase (Complex I) activity, sections were incubated at 37 °C for 1 h with the following incubation medium: 2 mM NADH, 0.6 mM nitroblue tetrazolium chloride (NBT) in 0.1 M phosphate buffer, pH 7.4. For Succinic dehydrogenase (Complex II) histochemical assay, sections were incubated at 37 °C for 60 min with the following incubation medium: 50 mM succinic acid, 1.5 mM NBT; 5 mM EDTA, 1 mM sodium azide, 1 mM 1-Methoxy-5-methylphenazinium methyl sulfate (mPMS) in 0.1 M phosphate buffer, pH 7.6. Control sections were incubated in absence of substrate. To stop the reaction, slides were washed twice for 5 min with PBS, mounted with a coverslip and immediately analyzed using a Zeiss Axio Scope.A1 microscope. The same acquisition settings were used in all experiments and groups. The mean grey value of OS and IS was determined in 16 ROIs, normalized to Control.

### 4.6. Oxygraphic Measurements

To measure the oxygen consumption from purified OS [71] from six–eight retina per group, an amperometric electrode (Unisense-Microsrespiration, Unisense A/S, Aarhus N, Denmark) was used. The experiment was performed in a closed chamber at 23 °C. Each sample (0.04 mg) was diluted 3:1 in ultrapure water, then added to the mixture by means of a Hamilton syringe, which granted a partial disruption of the OS, allowing substrates to permeate. Incubation medium was: 50 mM HEPES, 100 mM KCl, 2 mM MgCl_2_, 5 mM KH_2_PO_4_, 25 μg/mL ampicillin and 0.3 mM di(adenosine-5′) penta-phosphate as adenylate kinase inhibitor, pH 7.3 [72]. The content of the chamber was continuously mixed by an electromagnetic stirrer. Measurements were conducted under uncoupled conditions adding nigericin (5 µM) and valinomycin (10 µM) to the mixture prior to the sample additions. Oxidative substrates were: 20 mM fumarate to stimulate Complex I-III-IV pathway and 20 mM succinate to stimulate Complex II-III-IV pathway.

### 4.7. Laser Microdissection of Photoreceptors

The cultivated, unfixed and snap frozen eyes were sectioned vertically at 25-µm thickness. Slides were fixed in ice cold 70% ethanol for 30 sec, washed shortly in ice cold DEPC-treated water, dehydrated twice for 1 min in ice cold 100% ethanol and air dried for 5–10 min. To cut out the photoreceptor layer (ONL, IS, OS), a laser dissection microscope was used (PALM Carl Zeiss with PALM Robo software). Only the central area of the retina (2/3 of the total length) was dissected and collected from each section, in total six–eight sections per sample.

### 4.8. RNA Isolation

RNA isolation of the dissected photoreceptors was done using the RNeasy Micro plus (Qiagen, Hilden, Germany) Kit according manufacturer’s instruction. The collected tissue from laser microdissection was dissolved in RLT buffer containing β-Mercaptoethanol. The integrity and concentration of isolated RNA were assessed on an Agilent 2100 Bioanalyzer (Agilent Technologies, Santa Clara, CA, USA).

### 4.9. Sequencing and Bioinformatic Analysis

Complete cDNA was synthesized from 5 µl total RNA using the SmartScribe reverse transcriptase (Takara Bio, Kusatsu, Japan) with a universally tailed poly-dT primer and a template switching oligo followed by amplification for 12 cycles with the Advantage 2 DNA Polymerase (Takara Bio, Kusatsu, Japan). After ultrasonic shearing (Covaris S2, Woburn, Massachusetts, USA), amplified cDNA samples were subjected to standard Illumina fragment library preparation using the NEBnext Ultra DNA library preparation chemistry (New England Biolabs, Ipswich, MA, USA). In brief, cDNA fragments were end-repaired, A-tailed and ligated to indexed Illumina Truseq adapters. Resulting libraries were PCR-amplified for 15 cycles using universal primers, purified using XP beads (Beckman Coulter, Brea, CA, USA) and then quantified with qPCR (KAPA Biosystems, Basel, Switzerland). Final libraries were equimolarly pooled and subjected to 75-bp-single-end sequencing on the Illumina HiSeq 2500 platform (Illumina Inc., San Diego, CA, USA), resulting in ~25–32 mio reads.

Reads were mapped to the mouse genome (version mm10) with GSNAP (PMID:20147302; v2016-06-30) and splice sites from Ensembl (version 81) as support. RNA-seq data quality was assessed with RNA-SeQC (PMID:22539670; v1.1.8). Uniquely mapped reads served as an input for obtaining gene counts with featureCounts (PMID:24227677; v1.5.0) and Ensembl gene annotations (version 81). Normalization for library size and identification of differentially expressed genes was done with the R package DESeq2 (PMID:2551628; v1.12.13). To reduce the impact of sex-specific variance in gene expression, two male samples were included. Additional sources of unwanted variation in the data were modeled with the R package sva (PMID:22257669; v3.20.0) and included in the experiment design. DESeq2 p-values were adjusted for multiple testing (Benjamini-Hochberg) and genes with an adjusted *p*-value < 0.1 were considered as differentially expressed. The heat map of differentially expressed genes was generated using http://heatmapper.ca/.

### 4.10. In-situ Hybridization

For ISH we used two PCR-generated probes for *cryaa* and *cryab*. Primers used for probe generation are listed in the supplement, Table S2. PCR fragments were cloned into TOPO^®^vector. Probes were linearized with HindIII and transcribed with T7 using DIG RNA Labeling Kit SP6/T7 (Roche, Basel, Switzerland). Control sense probes were linearized with XhoI and transcribed with SP6 using DIG RNA Labeling Kit SP6/T7 (Roche 11 175 025 910, Basel, Switzerland). PFA fixed cryosections (14 µm) were washed two times with PBST (DEPC treated 1xPBS + 0.2% Tween 20) and pre-hybridized with hybridization solution (50% formamide, 5× SSC, 1× Denharts, 0.2 mg/mL Yeast RNA, 0.1 mg/mL Heparin, 0.1% CHAPS, 0.01 M EDTA, 0.4% Tween-20), for 1 h at 70 °C. Probes were diluted in hybridization solution, added to the slides and incubated at 70 °C over night. Unhybridized RNA was removed by washing twice with hybridization solution at 70 °C for 30 min followed by washing twice with MABT-buffer (100 mM maleic acid, 150 mM NaCl, 0.1% Tween-20). Slides were blocked with blocking solution (MABT + 1% blocking reagent: 1096176 Roche, Basel, Switzerland) for 1 h followed by incubation with anti-DIG antibody (11093274910 Roche, 1:4000 in blocking solution) over night at 4 °C. Slides were washed 5× 20 min with MABT-buffer and twice 10 min with NTMT-buffer (0.1 M NaCl, 0.1 M Tris HCl pH 9.5, 0.05 M MgCl2, 0.5% Tween-20). Signal detection was performed using 4-Nitro blue tetrazolium chloride/5-bromo-4-chloro-3-indolyl-phosphate (NBT/BCIP) according manufacturer’s instruction. To stop the reaction, slides were washed in 1 mM EDTA-PBS, mounted with a coverslip and analyzed using a Zeiss Axio Scope.A1 microscope. The same acquisition settings were used in all experiments and groups.

### 4.11. Further Analyses

Further analyses such as the TUNEL assay, immunohistochemistry and western blot analysis were performed according to established standard protocols and are listed in the Supplementary Materials and Methods.

### 4.12. Statistical Analysis

Values are presented as mean ± standard error of the mean (SEM). All statistical analyses were performed utilizing GraphPad Prism 5.03. One-way ANOVA and Bonferroni post hoc test was used to compare the groups. The value *p* < 0.05 was considered to be statistically significant.

## Supplementary Materials

Supplementary materials can be found at https://www.mdpi.com/1422-0067/21/7/2370/s1.

## Abbreviations

AMD	Age-related macular degeneration
BL	Blue light
C	Control
CCO	Cytochrome c oxidase
DCFDA	2′,7′–dichlorofluorescin diacetate
DEG	Differentially expressed genes
ETC	Electron transport chain
GCL	Ganglion cell layer
INL	Inner nuclear layer
IS	Inner segments
LCM	Laser capture microdissection
NIRL	Near-infrared light
ONL	Outer nuclear layer
ONL	Outer nuclear layer
OS	Outer segments
OXPHOS	Oxidative phosphorylation
PBM	Photobiomodulation
RL	Red light
ROI	Region of interest
ROS	Reactive oxygen species
RPE	Retinal pigment epithelium
SEM	Standard error of the mean
TUNEL	Terminal deoxynucleotidyl transferase (TdT) dUTP Nick-End Labeling
