# Supplementary material for: Photobiomodulation Mediates Neuroprotection against Blue Light Induced Retinal Photoreceptor Degeneration
Source: Int J Mol Sci. 2020 Mar 30;21(7):2370. doi: 10.3390/ijms21072370 (PMC7177783; doi:10.3390/ijms21072370)
Supplement: Supplementary file 1 [file ijms-21-02370-s001.zip › 20200319 Supplement Mol sci Heinig.docx]

**Supplement**

Photobiomodulation mediates neuroprotection against blue light induced retinal photoreceptor degeneration

**Nora Heinig^1^*, Ulrike Schumann^1^, Daniela Calzia^2^, Isabella Panfoli^2^, Marius Ader^3^, Mirko H. H. Schmidt^1,4,5^, Richard H. W. Funk^1^, Cora Roehlecke^1^**

^1^Institute of Anatomy, Medical Faculty Carl Gustav Carus, Technische Universität (TU) Dresden, School of Medicine, 01307 Dresden, Germany. nora.heinig@tu-dresden.de (N.H.), ulrike.schumann@tu-dresden.de (U.S.), mhhs@mailbox.tu-dresden.de, (M.S.), richard.funk@tu-dresden.de (R.F.), cora.roehlecke@tu-dresden.de (C.R.)

^2^Department of Pharmacy-DIFAR, Biochemistry and Physiology Lab., University of Genoa, Genova, Italy [panfoli@difar.unige.it](mailto:panfoli@difar.unige.it) (I.P.), dcalzia@gmail.com (D.C.)

^3^Center for Regenerative Therapies Dresden (CRTD), TU Dresden, Dresden, Germany marius.ader@tu-dresden.de (M.A.)

^4^ German Cancer Consortium (DKTK), Partner Site Dresden, Germany

^5^ German Cancer Research center (DKFZ), Heidelberg, Germany

*Correspondence should be addressed to: nora.heinig@tu-dresden.de

**Supplemental Materials & Methods**

Table S2: PCR primer for *in-situ hybridization* probe generation

|  | sequence 5‘-3‘ | PCR product (bp) |
| --- | --- | --- |
| Cryaa_for | GGACGTCACCATTCAGCATCC | 455 |
| Cryaa_rev | CATTGGAAGGCAGACGGTAGC |  |
| Cryab_for | TCTTCCCCTTCCACTCCCCAA | 444 |
| Cryab_rev | TGATGGGAATGGTGCGCTCAG |  |

*Immunohistochemistry*

Cultivated eyes were fixed in 4 % paraformaldehyde (PFA) at 4 °C overnight. For frozen sections, tissue was incubated in 30 % sucrose solution cryoprotected for 3 h. Next, the eyes were embedded in Tissue Tek OCT Compound, snapped frozen in dry ice and cryosectioned at 14 µm thickness in the vertical plane. For paraffin sections, eyes were dehydrated and embedded using a standard protocol. Next, the eyes were sectioned at 5 µm thickness in the vertical plane.

For paraffin sections, eyes were dehydrated at room temperature (RT) for 2x 45 min in 70 % (v/v) EtOH, 2x 45 min in 96 % (v/v) EtOH, 2x 45 min in EtOH 100 % (v/v) and 30 min in xylene and 2x 45min paraffin at 65 °C. After embedding in paraffin the eyes were sectioned at 5 µm thickness in the sagittal plane. Flattened sections were mounted on pre-silanized slides, dried overnight at 37 °C and stored at RT. The slides were deparaffinized and cooked 2x5 min in citrate buffer pH 6.0 if antigen retrieval was recommended.

Frozen- and paraffin-sections were washed 2x 5 min in PBS buffer and blocked in blocking solution (PBS+5 % normal goat serum+0.3 % Triton X 100 + 1 % bovine serum albumine (BSA) for 1 h at RT. The primary antibody was incubated overnight in a humid chamber at 4 °C. Slides were washed 2x 5 min in PBS and incubated with the secondary antibody + DAPI for 2 h at RT. Slides were washed 2x 5 min in PBS and a coverslip was mounted. Fluorescence images were obtained using the microscope Zeiss Observer Z.1 with Apotome with the same acquisition settings throughout the different groups.

The following antibodies were used for immunohistochemistry: polyclonal rabbit anti-Nox-4 (ab60940; Abcam, 1∶200), polyclonal rabbit anti-gp91-phox ( = anti-Nox-2; 07-024; Millipore, 1:50), monoclonal mouse anti-4HNE (MAB6115; Abnova; clone HNEJ-2, 1∶20), monoclonal mouse Hexanoyl-Lys [HEL], (MHL-021P, clone 5F12, biomol, 1:70), polyclonal rabbit anti-Caspase-9 (NB100-56118, Novusbio, 1:700), mouse monoclonal anti-Cryαa (sc-28306, Santa Cruz, clone B-2, 1:150), polyclonal rabbit anti-Cryαb (ADI-SPA-225-E, Enzo, 1:150), monoclonal mouse anti-Caspase-3 (610322, BD, clone CPP32, 1:150), polyclonal rabbit anti-Bax (sc526, Santa Cruz, 1:300), monoclonal mouse anti-Bcl-2 (2207, Immunotech, 1:100), polyclonal goat anti-Glutamine Synthetase (sc6640, Santa Cruz, 1:200); secondary antibodies: Anti-Rabbit IgG,F(ab’)2 Fragment & Anti-mouse IgG (H+L),F(ab’)2 Fragment (Alexa Fluor 488, Alexa Fluor 555, Cell Signaling, 1:500), Anti-goat IgG (Alexa Fluor 488, Invitrogen, 1:100).

*Western blot analysis*

To prepare protein lysates of complete retinas, for each sample 2-4 dissected retinas subjected to the same treatment were pooled together in PBS containing cOmpleteTM Protease Inhibitor Cocktail. Then, retinas were incubated for 10 min in ice cold RIPA lysis buffer and homogenized with an ultrasonicator. Next, the samples were incubated on ice for 10 min. After centrifugation at 8000 g for 10 min at 4 °C, supernatants were collected. Preparation of OS protein was done according to the protocols (Burns et al., 2006) from 8 dissected and pooled mouse retinas for each sample.

Total protein of the lysate supernatant was determined using BCA Protein Assay Kit (Thermo Scientific). Protein lysates were mixed with 6x Laemmli buffer and incubated at 95 °C for 5 min. Per lane, 5 µg of protein was loaded on a 10 % SDS-polyacrylamide gel. The separated proteins were transferred to a 0.45 µm PVDF-membrane (Immobilon-P™; Millipore). After blocking the membrane in blocking solution for 1 h it was incubated with primary antibody overnight at 4 °C. The membrane was washed 3x 10 min with TBST, probed for 1 h with secondary HRP conjugated antibody and washed 3x 10 min in TBST. The bound antibody was detected in an Image Reader LAS-3000 (Fuji Photo Film) using the Immobilon Western Chemiluminescent HRP Substrate (Millipore) according to the manufacturer’s instructions. Briefly, 5 ml peroxide solution and 5 ml Luminol reagent were mixed in a 15 ml tube protected from light. Membranes were incubated in the mix for 3 min, transferred to a plastic foil and the chemiluminescent reaction was detected in an Image Reader LAS-3000 (Fuji Photo Film) at exposure times with 10 sec increments.

Protein quantification was performed with ImageJ free software. Expression level of the protein of interest was determined relative to their loading control (β-actin). Values were normalized to control sample (n ≥ 4 experiments). Only unsaturated bands of blots were used for quantification.

The following antibodies were used for western blot analysis: Monoclonal mouse anti-OXPHOS Rodent WB Antibody Cocktail (ab110413, Abcam, 1:800, contains 5 antibodies against the 5 OXPHOS complexes. Complex I: NDUFB8, Complex II: SDHB, Complex III: UQCRC2, Complex IV: MTCOI, Complex V: ATP5A); polyclonal rabbit anti-Caspase-9 (NB100-56118, Novusbio, 1:500), monoclonal mouse anti-Bcl-2 (Cat. 2207, Immunotech, 1:500), polyclonal rabbit anti-Bax (sc526, Santa Cruz, 1:1000), polyclonal rabbit anti-Nox-4 (ab60940; Abcam, 1∶250), polyclonal rabbit anti-β actin (NB100-78420; Novus Biologicals, 1∶1000), secondary HRP conjugated antibodies: Anti rabbit IgG (Thermo Fisher Scientific, 1:10000), Anti-mouse IgG (Cell Signaling Technology, 1:2000); Anti-goat IgG (Jackson Immuno Research, 1:3000).

*TUNEL assay*

TUNEL assay was used to detect fragmented DNA in irradiated photoreceptor cells (In Situ Cell Death Detection Kit TMR red; Roche Diagnostics). The fixed and paraffin embedded eyes were sectioned at 5 µm thickness. Slides were stained according manufacturer’s instruction and nuclei were counterstained with DAPI. Mosaic fluorescence images of the whole retina were obtained using a Zeiss Observer Z.1 with Apotome. Same acquisition settings were used in all experiments and groups. Cells from the outer nuclear layer (ONL) were counted automatically with ImageJ software (http://imagej.nih.gov/ij/). For counting TUNEL+ and total number of cells a fixed threshold for DAPI channel and TUNEL+ channel was adjusted for all sections. Only the central area of the retina was counted, 2/3 of the total length. Peripheral parts were removed because blue light effects basically the central part of the eye (Roehlecke et al., 2011).

**Supplemental Figures**

**
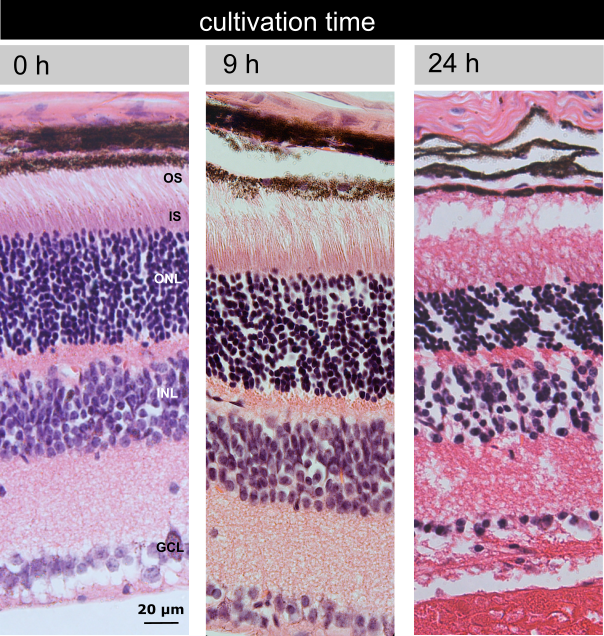
**

**Fig. S1: Representative images of HE-staining in retinal sections for different cultivation periods.**

Eyes were cultured for 9 h or 24 h or directly fixed without cultivation (0 h). Retinal structure is nearly unaffected after 9 h of cultivation. Retinal layers: retinal pigment epithelium [RPE], outer segments [OS], inner segments [IS], outer nuclear layer [ONL], inner nuclear layer [INL], ganglion cell layer [GCL]. N=3 experiments.

**
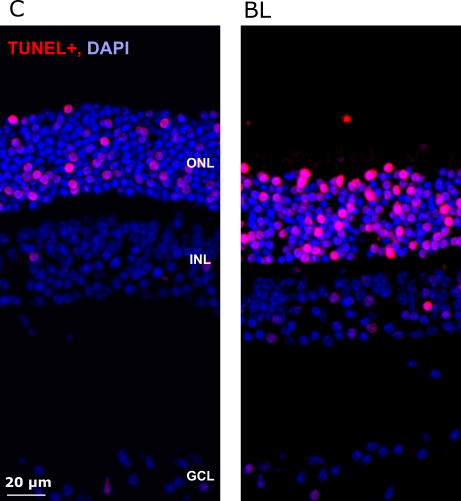
**

**Fig. S2: Blue light causes damage especially in photoreceptors.**

Eyes were cultured for 9 h, either not damaged (C-Control) or damaged for 6 h with blue light (BL). Representative images of terminal deoxynucleotidyl transferase-mediated deoxyuridine triphosphate (dUTP) nick end labeling (TUNEL)-assay labeled retinal sections. Staining demonstrates cell death through labeling DNA strand breaks. Red represents cells with DNA strand breaks labeled with tetramethylrhodamine (TMR)- dUTP, blue represents DAPI- stained nuclei. Photoreceptors are predominantly damaged by blue light whereas cells of INL and GCL remain mainly unaffected. Retinal layers: outer nuclear layer [ONL], inner nuclear layer [INL], ganglion cell layer [GCL]

**
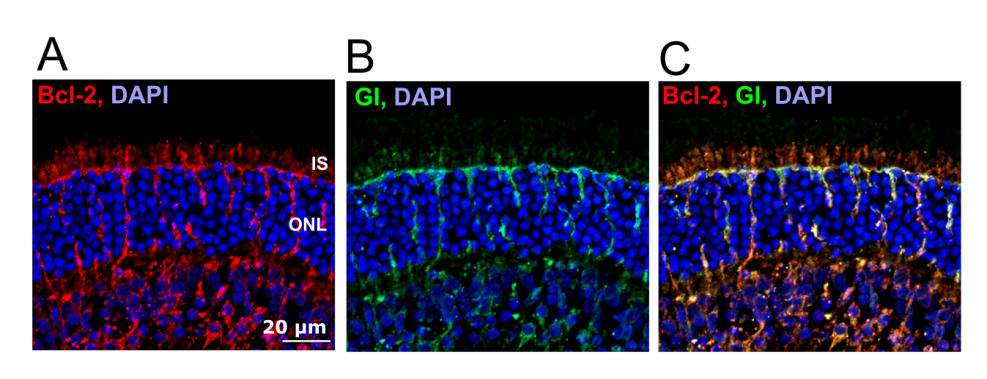
Fig. S3: Bcl-2 is localized in photoreceptors IS and Müller cells.**

Representative images of Bcl-2 and Glutamine Synthetase (GI) immunohistochemical staining in retinal sections. Retinal layers: inner segments [IS], outer nuclear layer [ONL]. **A:** Bcl-2 expression. **B:** GI expression in Müller cells. **C:** Bcl-2 expression colocalized with GI in the photoreceptor IS and Müller cells.

**
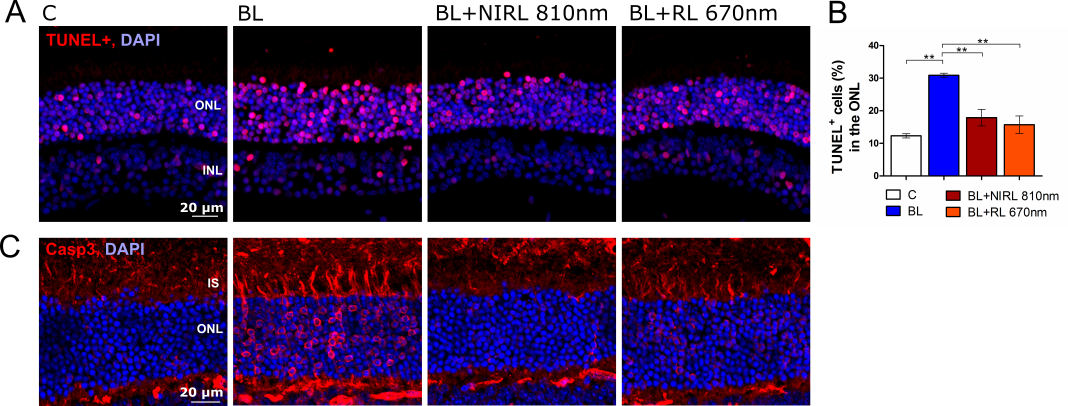
**

**Fig. S4: NIRL and RL induce increased photoreceptor survival in BL irradiated cells.**

Late event analysis after 9 h of cultivation. Eyes were treated for 6 h with BL, following 10 min NIRL or RL and post-cultivated for another 3 h. A: Representative images of terminal deoxynucleotidyl transferase-mediated deoxyuridine triphosphate (dUTP) nick end labeling (TUNEL)-assay labeled retinal sections. Staining demonstrates photoreceptor cell death through labeling DNA strand breaks. Red represents cells with DNA strand breaks labeled with tetramethylrhodamine (TMR)- dUTP, blue represents DAPI- stained nuclei. **B:** Quantitative analysis of TUNEL-positive cells. The graph displays the ratio of TUNEL-positive to total number of cells in the ONL. Central part of the retina (2/3 of total length) was counted. N=3 experiments ± SEM. **P<0.01 compared with BL. **C:** Representative images of Caspase-3 (Casp3) immunohistochemically staining in retinal sections. N=3 experiments. Retinal layers: inner segments [IS], outer nuclear layer [ONL], inner nuclear layer [INL].





**Fig S5: NIRL and RL do not change the retinal mRNA expression of NADPH oxidase Nox-4 in BL irradiated cells.**

Late event analysis after 9 h of cultivation. Eyes were treated for 6 h with BL, following 10 min NIRL or RL and post-cultivated for another 3 h. Relative mRNA expression of Nox-4 in the retinas compared to time- matched non-irradiated control. The mRNA expression was quantified by real-time PCR. Rpl32 was used as reference gene. N=5 experiments ± SEM.

**
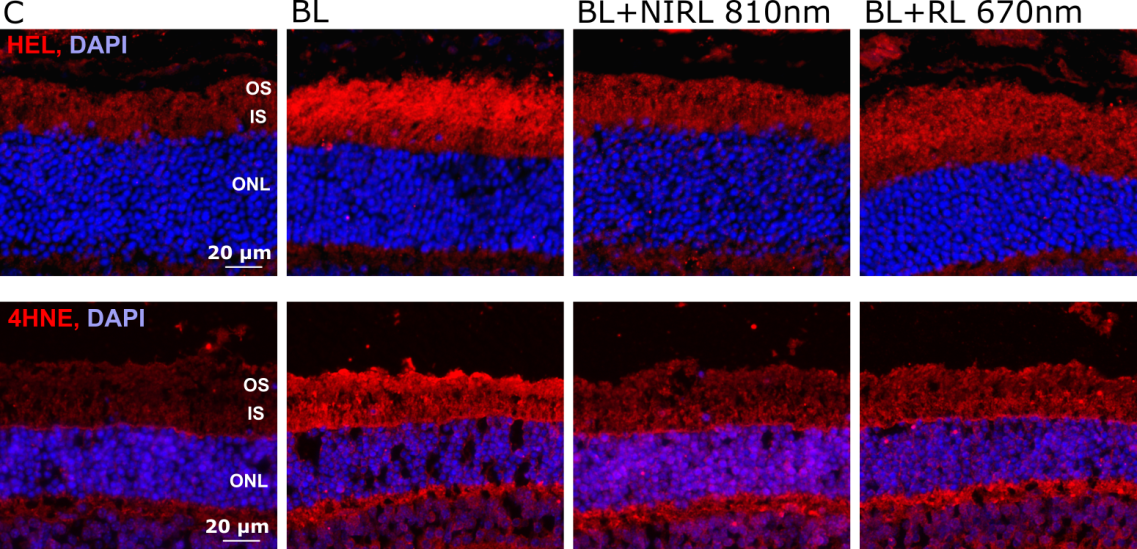
**

**Fig. S6: NIRL and RL decrease lipid peroxidation in BL irradiated cells.**

Representative images of HEL and 4HNE immunohistochemical staining in retinal sections. Late event analysis after 9 h of cultivation. Eyes were treated for 6 h with BL, following 10 min NIRL or RL and post-cultivated for another 3 h. Retinal layers: outer segments [OS], inner segments [IS], outer nuclear layer [ONL]. N=3 experiments.


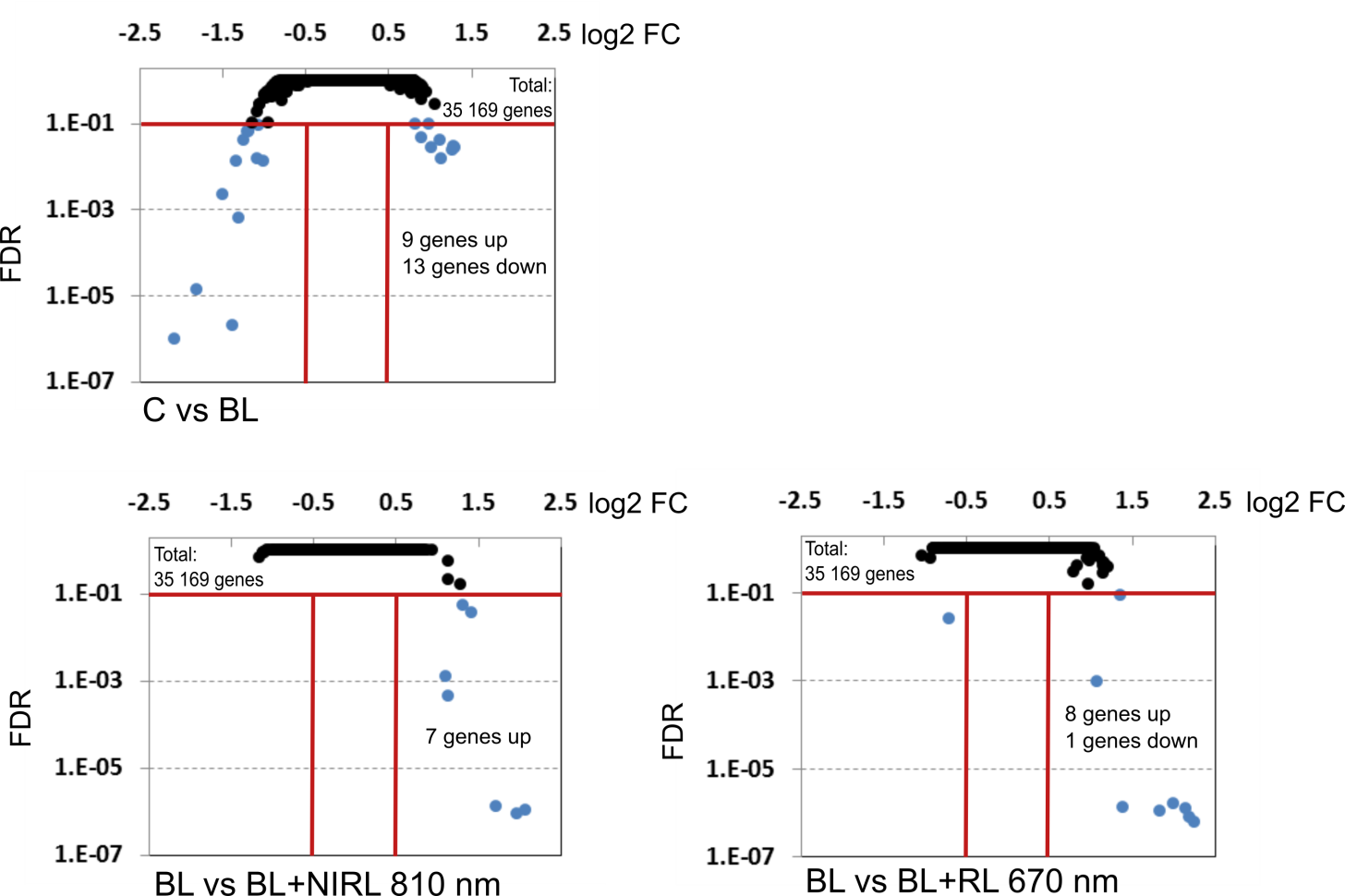


**Fig. S7: NIRL and RL regulated gene expression analysis of isolated photoreceptors illustrated in volcano plots.** Late event analysis after 9 h of cultivation. Eyes were treated for 6 h with BL, following 10 min NIRL or RL and post-cultivated for another 3 h. Volcano plots showing annotated genes and significant differently expressed genes (DEGs) from C_BL, BL_BL+NIRL 810 nm and BL_BL+RL 670 nm. Genes with significantly differential expression (false discovery rate FDR <0.1) which are either up or down-regulated are highlighted in blue.


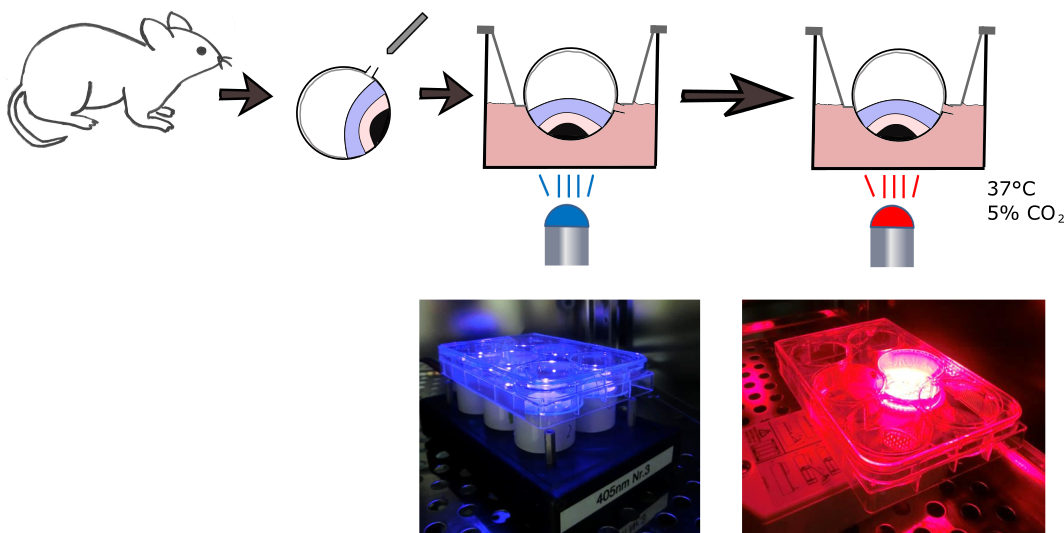


**Fig. S8: Schematic overview of mouse *ex vivo* mouse eye cultivation with light treatment.**

Murine eyes were removed from the orbit, positioned in a cell culture insert, faced with their cornea to the light diodes and cultured in medium for 9 h in 37 °C and 5 % CO_2._

**Supplemental full length western blots**

**
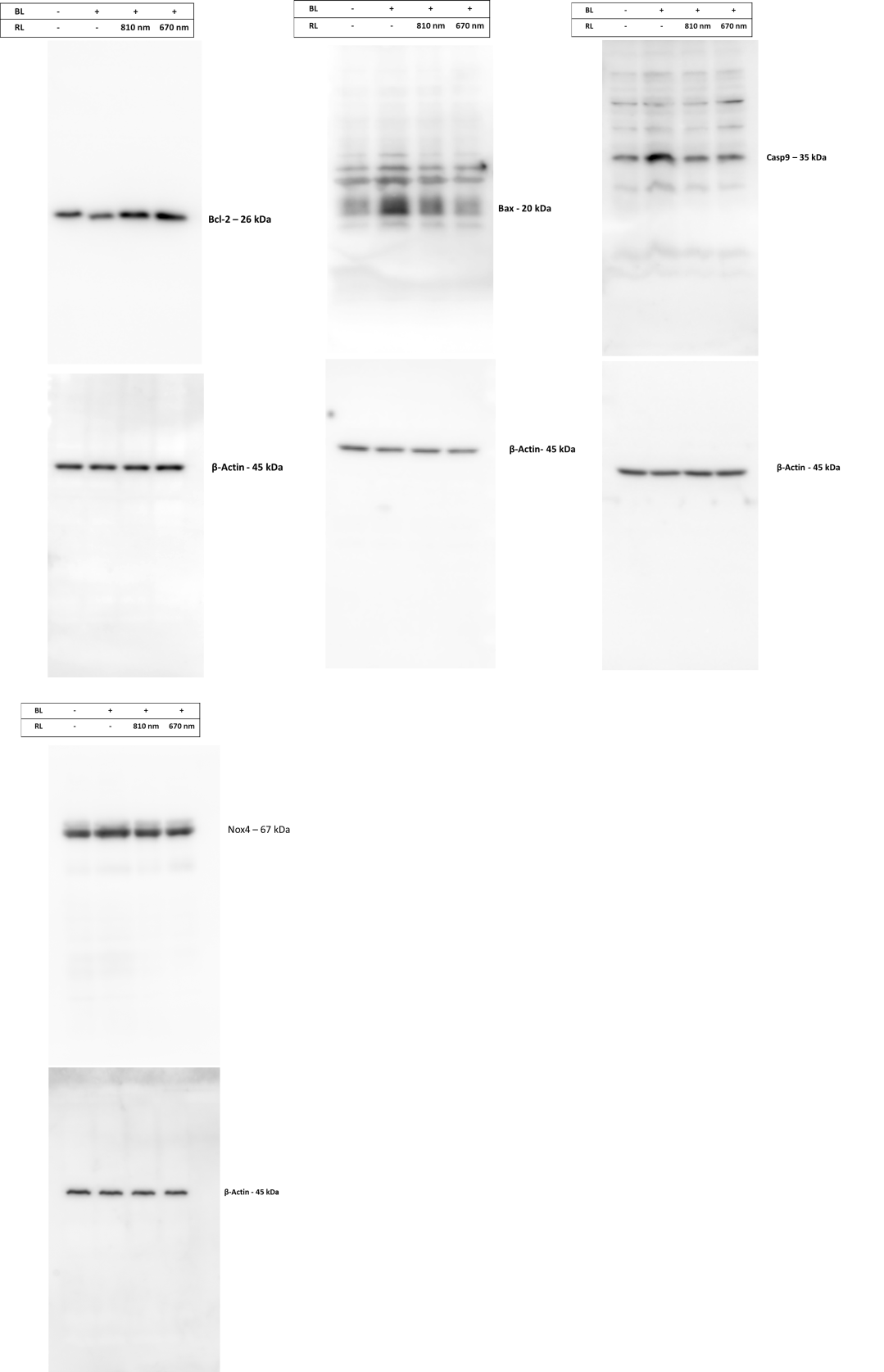
**Representative full length western blots of cropped western blots shown in the paper figure 2, figure 3, figure 4.

**
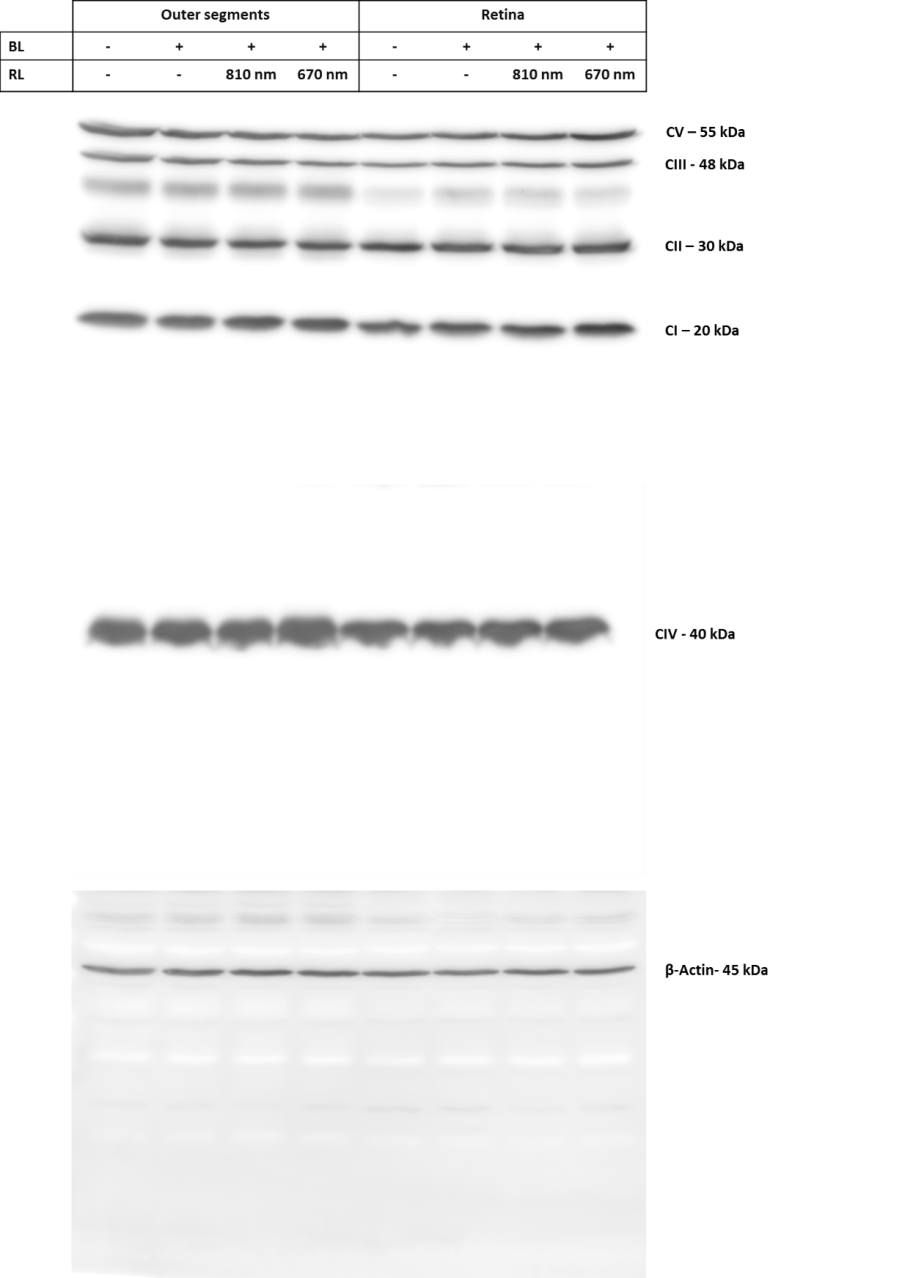
**
